# Discharge after coronary artery bypass grafting: A qualitative study highlighting the need for individualised, person-centred care

**DOI:** 10.1371/journal.pone.0349948

**Published:** 2026-06-17

**Authors:** Melanie Rushton, Michelle Howarth, Heather Iles-Smith

**Affiliations:** 1 School of Health and Society, University of Salford, United Kingdom; 2 Department of Health and Social Care at Edge Hill University, United Kingdom; Mazandaran University of Medical Sciences, IRAN, ISLAMIC REPUBLIC OF

## Abstract

**Background:**

This study explores patient preparedness and support during discharge following coronary artery bypass graft (CABG) surgery. Despite the importance of effective discharge planning, many patients feel unprepared and unsupported, which can hinder recovery and increase readmission risk.

**Aim:**

To examine patient experiences of the discharge process post-CABG, focusing on education received and perceptions of person-centred discharge planning.

**Methods:**

A qualitative study using constructivist grounded theory was conducted with eleven participants, interviewed 4–6 weeks post-surgery at a UK Northern NHS hospital. Semi-structured interviews were digitally recorded, transcribed, and analysed using a constant comparative method. Recruitment, data collection, and analysis occurred concurrently. Despite the modest sample size, the study achieved strong information power as the research aim was narrow and clearly focused on exploring the lived experiences of patients who had had a coronary artery bypass graft. This allowed for in-depth engagement with each participant, with relevant experience, enhancing the relevance and the richness of the data. Thematic saturation was observed early in the analysis, indicating that the data sufficiently addressed the research questions. The use of a rigorous analytical approach further ensured that the insights drawn were both meaningful and robust, supporting the adequacy of the sample size. The COREQ checklist guided reporting.

**Results:**

Four key themes emerged: Patients avoided seeking support or “making a fuss.” Many felt unprepared for discharge. The information booklet was a valuable resource. Lack of post-discharge contact and continuity caused concern. Some participants reported insufficient pre-discharge information and unclear pathways for post-discharge support. The booklet was often the primary source of guidance.

The participants’ reluctance to seek support could reflect a pattern of self-management shaped by uncertainty, low confidence, a desire not to burden healthcare professionals or a belief that doctors know best. Yet many felt unprepared for discharge perhaps highlighting gaps in communication and discharge planning. There also seems a reliance on written materials, such as the information booklet. Limited post- discharge contact, created further anxiety, where patients were unsure where to turn for advice.

These findings point to the need for clearer, more person‑centred discharge processes. Better communication, improved pre‑discharge education, and clear pathways for follow‑up support could strengthen patient confidence and safety. Although booklets are helpful, they should supplement and not replace ongoing contact and personalised guidance. Ensuring continuity of care and enabling patients to seek help without hesitation is essential for effective person‑centred discharge practice.

**Conclusion:**

Findings highlight the importance of person-centred approaches in discharge planning. Tailored education and support could improve patient confidence and symptom management during recovery, potentially reducing readmission rates.

## Introduction

Coronary heart disease (CHD) is a global concern [1] and is acknowledged to be a leading cause of death in many countries [2]. Typical treatment for improving cardiac circulation includes procedures such as a coronary artery bypass graft (CABG). CABG is a common procedure with 160,000 isolated CABG surgeries in the United States reported in 2019 [3]. In the UK The National Adult Cardiac Surgery Audit (NACSA) [4] part of the National cardiac audit programme (NCAP), reported 14,527 isolated non-emergency CABG procedures in 2017/18. More recently, in 2022/23 NACSA reported a decline in the number of procedures to almost 4000 operations per year. Despite the reduction in procedures there are still significant numbers of people requiring this life-changing and life-saving procedure. The success of this clinical intervention is recognized globally. For example, in Canada, Habib et al (2015) [5], reported that CABG has successful results in terms of survival and reduced need for reintervention compared to percutaneous coronary intervention (PCI) (4-year mortality 16.4% CABG compared to 20.8% PCI). CABG is acknowledged as the superior treatment, offering substantial survival benefits and reduction in further myocardial infarction in patients with multivessel disease [6].

Improving the quality of life for patients has been at the vanguard of contemporary practice, and modern health care has been challenged to advance service provision to one that is patient-centred and takes account of individual preferences and beliefs. Person-centred care (PCC) is central to good quality health care and services [7], although in practice this can be challenging [8]. In the UK, the NHS long term plan (2019) [9] identified the need to shift how healthcare works alongside patients to deliver more person-centred care, recognising ‘what matters to someone’ is not just ‘what is the matter with someone’. A person-centred approach puts people at the heart of health and social services, including care, support, and enablement, encouraging individuals to play an active role in their care [9]. According to Park et al. (2018) [10], PCC interventions for patients entails patient empowerment, physical support, and information provision; for healthcare professionals focusing mainly on education, training and improvement of the continuity and coordination of care. In the UK the Nursing and Midwifery Council places a greater emphasis on PCC, and in 2024 the NMC launched the, ‘seeing the whole person supports better care’ campaign, which supports nurses to continue to think holistically when providing care to see the whole person [11].

Although PCC was first discussed by Picker over four decades ago and prior to this ‘care for the entire self’ was described in the late 1950’s and 60’s in the context of psychiatry by Rogers, 1995 [12], yet it is not always applied in practice [13]. The Picker Institute was established in 1986 in the USA, a non-profit organization dedicated to developing and promoting a patient-centred approach to healthcare (Picker Institute, 2024) [14] Despite the wealth of literature and longevity of PCC in health care, still little is known about the application of PCC during the care of CABG patients, particularly related to the discharge process.

Globally, person-centred care is gaining widespread acceptance, and Goldfarb et al (2024) [15] identify that the American Heart Association, (2024) increasingly recognises it as improving patient engagement, shared decision making, and promoting personalised and effective cardiovascular care. The European Society of Cardiology (ESC), (2023) [16] identified a focus on person centred healthcare as one of their six strategic aims in their strategic plan 2023–2028, emphasising the patient perspective in education.

In the UK, person centred care (also referred to as patient centred care) is fundamental in healthcare training, service delivery and planning [17]. The focus is to provide care that supports patients’ values and needs, empowering patients and providing personalised care. A range of work is currently being undertaken to develop new ways of working with the aim of moving away from disease-based care delivery to one that embraces a more patient centred model of working. A recent shift towards this resulted in the NHS [18] providing person centred care delivery training, designed by Skills for Health (2017) [19]. This training includes six principles to develop the workforce with the behaviours, skills and competencies that support, and drive person centred approaches to well-being, prevention, care and support. The six principles require that care is person centred, services are created in partnership, focus is on equality and narrowing inequalities, involving carers, as well as voluntary, community, social enterprise and housing sectors. Key partners, volunteers, and social action are recognised as key enablers.

Significantly, conversations with service users and carers indicates that care may not always be patient-centred, and a gap persists particularly in relation to pre-assessment and post-recovery of surgical procedures. Byrne et al. (2020) [13] discuss the theory/ practice gaps apparent in the challenge of translating the ideas of PCC into a concrete concept. In their Australian based review, they report nurses as seeing PCC as ‘extra’ or additional to their roles even though PCC aligns with the Registered Nurse Standards for Practice in Australia, and an intrinsic element of the nursing profession. Alternatively, Kwame & Petruka, (2021) [20] identified communication as one of the key barriers for patient centred care in nurse interventions, and that patient centred communication is fundamental to ensuring optimal health outcomes.

Patient-centred engagement is essential for a successful discharge and recovery period; conversely, disengagement from patient-centred conversations and education can have a significant effect on the confidence of the patient to effectively manage their recovery both mentally and physically [21]. The need for patient-centred approaches was also reported by Kuipers et al. (2021) [8], this qualitative study in the Netherlands, revealed that co–creation of care, through inclusion in decision making, positively influenced patient satisfaction and wellbeing. This approach is echoed in the UK’s NHS Improvement framework which advocates that discharge planning should begin at first contact between patient and the health care system and continue through to discharge. [18]. To date there is little evidence describing the patients journey during the discharge process following CABG. This limits the clinicians understanding of the patients’ needs and provides reduced opportunity to implement person centred care.

This study aims to explore CABG patient’s perspective and lived experience of the discharge process and understand their perceptions of person-centred discharge planning and related information and education provision.

## Methods

### Design

This qualitative study used an adapted constructivist grounded theory approach [22] to capture meaningful, subjective data and provide an insight into the participants experience of person-centred discharge planning and education following non-urgent CABG surgery.

Constructivist grounded theory allows the generation of theory through a systematic, iterative, and rigorous data collection and analysis process [22]. This type of research looks at how people interpret their experiences and perspectives and uses these interpretations to develop a deeper understanding of the topic under study.

The primary objective of this study was not to develop a formal theory, but the adapted constructivist grounded theory approach was selected because of its methodological suitability for examining complex phenomena such as discharge experience following CABG as it allows a systematic and iterative analytical structure, through constant comparison, layered coding and memo writing.

In depth one-to-one interviews were conducted with participants on one occasion 2–5 weeks post-surgery. Concurrent participant selection, recruitment, data collection and analysis, allowed us to gather data to answer questions from the emerging analysis [22]. The data collection period spanned from 20/03/2018 to 21/06/2019. As data were collected between 2018 and 2019, readers should consider the potential temporal gap when interpreting the findings from the study, as cardiac services and discharge practices have evolved in subsequent years inclusive of digital health practices.

### Study setting, sample and participants

Participants were recruited from a hospital in the Northwest of England where patients were admitted for CABG surgery. Potential participants were approached in clinic prior to surgery and provided verbal and written descriptions of the interviews/study (that would take place following discharge). Participant informed written consent was obtained by the clinician in the preoperative assessment clinic to allow the researchers to contact them after their surgery and seek further consent regarding participating in the interview. All patients who fit the inclusion criteria were asked in the preoperative assessment clinic if they wanted to be part of the research with a full explanation and consent obtained for the researchers to contact on discharge. However, those patients who declined consent at any part of the process were not included in the study, this did not affect their treatment plan

Once discharged home potential participants were telephoned by the research team to seek verbal consent to participate in the study and arrange the home interview. We recognized that the recruitment process involved power imbalances, as patients were approached by a member of the preoperative assessment clinic team in a pre-surgical context. This may have influenced patients’ perceived ability to decline participation. To mitigate this voluntariness was emphasised, we provided time for them to decide. The researchers who contacted them were not involved in their care, it was made clear that declining would not affect treatment. The interview timeline (two to five weeks post discharge) was chosen to maximise accurate recall while allowing initial recovery and preventing memory decay. The 2–5-week period was sufficiently recent to aide memory recall whilst providing time for recovery and to recover from major surgery. Additionally, cardiac rehabilitation commonly begins at 6 weeks and therefore, interviewing before this time reduces the prospect of the participants experiences being influenced. The timing helps maintain internal validity, ensuring that insights relate genuinely to the early recovery phase rather than to formal rehabilitation content.

### Inclusion/ exclusion criteria

Inclusion and exclusion criteria (Table 1) was used to help guide participant recruitment. Importantly sampling was guided by a constructivist grounded theoretical framework to enable inductive reasoning [23]and therefore theoretical sampling was used to recruit five to 24 participants in line with Hennick & Kaiser (2022) [24] suggestion for qualitative research.

**Table 1:** Inclusion / Exclusion criteria.

Inclusion	Exclusion
Male or female over 18 years of age	Cardiac surgery other than CABG
Ability to provide informed consent	Inability to provide informed consent
Experience of first non-urgent isolated (not including any other procedures) CABG.	Non isolated CABG, i.e., combined valve surgery
English speaking (no funding for interpreter)	People unable to understand or speak English
Able to provide written informed consent	Patients with cognitive limitations

In our study, we used theoretical sampling alongside constant comparative recruitment, data collection and analysis until information power led to emergent themes (that is sufficient power of information held by the sample) and/or no new insights were gained from additional data. Despite the modest sample size (11 participants), the study achieved strong information power (Hennink & Kaiser 2022) as the research aim was narrow and clearly focused on exploring the lived experiences of patients who had had a coronary artery bypass graft. This allowed for in-depth engagement with each participant, with relevant experience, enhancing the relevance and the richness of the data. The use of a rigorous analytical approach further ensured that the insights drawn were both meaningful and robust, supporting the adequacy of the sample size. In this study, sample adequacy was evaluated using information power rather than relying solely on thematic saturation. Information power considers the relevance, specificity and depth of data in relation to the study aim. The focused research question and richness of participant narratives ensured that information power was sufficient without necessitating complete thematic redundancy.

### Data collection

Data were collected by experienced nurse academic researchers MR and MH both white females, the first, a cardiac nurse with a MA in Education Research and the second a registered nurse with a PhD, both employed as academics which may have influenced how interviews were conducted and interpreted.

During telephone contact all participants provided informed verbal consent to take part in a face-to-face interview in their own home. Before conducting home-based interviews, all participants had an information sheet outlining the purpose of study, procedures, voluntary participation and the right to withdraw. At the scheduled home visit the researcher verbally reiterated the information, answered the participants’ questions and confirmed that the participation had fully understood the study. Written informed consent was obtained before the interview began, which included consent to the audio recording of the interview. Participants were reminded that they could withdraw or skip questions without providing a reason. The Informed written consent was securely stored in accordance with institutional data protection policies. The safety of the researcher was ensured by carrying a mobile phone, and the data collector informed a colleague of the address and time of entry and leaving.

The initial interview schedule (Table 2) was developed based on Pickers Institute 8 key principles of person-centred care (Table 3), in collaboration with a service user group and piloted with members of the group. Adaptations were made to the schedule prior to further interviews and adapted as interviews progressed based on emerging findings. Data from the two pilot interviews was deemed to be of sufficient depth and quality to be included within the final analysis. Interviews were digitally recorded and transcribed verbatim by Outsec transcription service [25].

**Table 2:** Interview schedule.

no.	Question	Picker institute principles
1	Please describe your experience of the day you left hospital.	Fast access to reliable healthcare advice
2	What level of involvement did you have? What information were you given (format, timing, content)?	Effective treatment by trusted professionals
3	Can you share your experiences of community follow-up after your discharge, and whether you found it helpful?	Continuity of care and smooth transitions
4	How did you feel about your experience after being discharged, and what steps, if any, did you take to reach out to healthcare staff?	Involvement and support for family and carers
5	What types of questions did you ask the staff?	Clear information, communication and support for self-care
6	What changes do you think should be implemented Discharge processes? and why do you think they would be beneficial?	Involvement in decisions and respect for preferences
7	How were you and your family/friends involved in the discharge planning?	Emotional support, empathy and respect
8	How did the discharge plans impact your feelings of anxiety, and in what ways did they help or not help?	Attention to physical and environmental needs

**Table 3:** Pickers 8 Principles of Person Centred care (The Picker Principles of Person Centred care - Picker) https://picker.org/who-we-are/the-picker-principles-of-person-centred-care/).

1	Fast access to reliable healthcare advice
2	Effective treatment by trusted professionals
3	Continuity of care and smooth transitions
4	Involvement and support for family and carers
5	Clear information, communication and support for self- care
6	Involvement in decisions and respect for preferences
7	Emotional support, empathy and respect
8	Attention to physical and environmental needs

At the end of the interview patients were also asked about their understanding of person-centred care with the following questions: -

1. How would you define person centred care?
2. In what ways were you involved in person centred care?

### Data analysis

Constant comparative analysis was used to analyze and interpret datum by continually comparing emerging data with existing data. This method helps to ensure information power by refining and validating themes from the interview data. The method of analysis uses an inductive approach, comparing data segments to identify patterns and themes as they emerge. Inter-rater reliability was assured through team members independently reviewing and analyzing the data using a structured analytic process to facilitate a constant comparative approach. The two researchers coded the data line by line through an iterative process to identify lower-level concepts and first stage codes. The in-vivo codes were formed using the participants’ own words to create a coding scheme for analysis. This provided greater understanding of the data gathered so far and informed further data collection. The interview schedule was adapted in response to prior analysis, and the knowledge from previous interviews allowed a change in direction of some of the questions to further explore findings [22]. Information power was reached when no new themes or information emerged and once the themes were fully formed [24].

### Ethical approval

Ethical approval was granted from our institution and NHS Health Research authority (14/NI/1155).

### Rigour

Validity and reliability were addressed with use of the COREQ (COnsolidated criteria for REporting Qualitative research) checklist to report the findings [26]. Interviews were analysed by two researchers (MR and MH), and any differences of opinion were resolved through discussion between the two analysts. The collaborative approach ensured rigour throughout the process.

## Results

A total of 11 (10 male) CABG patients, between 52 and 72 years, participated in the study over a period of 2 years. The sample was predominantly male as the pool of eligible participants reflected the clinical population undergoing CABG surgery. Epidemiologically, men are significantly more likely to develop coronary artery disease (CAD) at a younger age requiring interventions. The majority of patients available for recruitment at the time were male, therefore the gender imbalance was a natural characteristic of the sampling rather than a recruitment bias.

Participants ethnicity was predominantly white British with just one being from an Asian background. Interviews lasted an average of 46 minutes (between 30.30 and 78.50 minutes).

Participants were keen to share details of their experiences of their journey from initial pre-operative assessment through to discharge. Their stories provided valuable insight into the patient journey, revealing four key themes:

1) not making a fuss/not seeking support: participants didn’t want to draw attention to themselves, didn’t want to make a fuss’and didn’t want to ‘mither’ (bother) the GP, and expressed doubts regarding what was necessary to seek help from healthcare professionals with some participants relying on the internet. (see Table 4)
2) feeling unprepared to be discharged home: participants described feeling unprepared for lifestyle changes in the recovery period and didn’t feel ready to be discharged home (see Table 4).
3) the information booklet was useful for providing guidance: participants found the information booklet (provided by the British Heart Foundation) useful because it helped them to prepare for the surgery and could subsequently refer to it on discharge, with some participants valuing information from other patients (Table 4).
4) a lack of contact/continuity to reassure the patient: some participants commented on the lack of contact from health care professionals once discharged from hospital (Table 4).

**Table 4:** Quotations from participants for each theme.

Table 4- Themes and verbatim comments:
Not making a fuss/ not seeking support. *‘That’s the key thing, are you making a fuss****’****?* ***(Participant 1).*** ‘*See how it goes*’:*‘My daughter came here, and she wanted to take me back to the hospital, ‘I’ll be alright. I’ll see how it goes...’* ***(Participant 2)***. *‘I put up with the pain, and it wasn’t that bad anyway*’ ***(Participant 3).*** *‘I did feel a little bit alone; I didn’t ring for support****’ (Participant 4).*** *‘So, I thought I’ll ring him rather than go to the doctor’s.... I’ve got his card......I could have rung them…. I thought I was just being a bit daft.,.***’ *(Participant 3).*** *‘You don’t really want to keep mithering the GP with things that are probably what you expect to have after an operation’* ***(Participant 6).*** *‘Pain-wise, feeling wise, anything I wasn’t sure about or wanted to know. I had to go on the internet to have a look myself.’* ***(Participant 6).***
Feeling unprepared to be discharged home. *‘Unprepared at the end’ (****Participant 7****).* *‘I don’t think I was involved. I think it was more of, ‘You can go today’, then, within half an hour or an hour of that, I was dressed and ready to go.****’ (Participant 7).*** *You are doing very well; I’ve looked at your records, and everything seems fine’* ***(Participant 1)***. *‘I really don’t remember being asked anything about discharge’* ***(Participant 8).*** *Felt quite vulnerable’* ***(Participant 4)***. ‘W*hen x came in and said you could go home; I did say to him I’d rather stay in an extra day if you don’t mind.* **(Participant 4).**
Information booklet was useful for providing guidance. ‘*The booklet…, it covered a whole range of stuff …the exercises you can do, …. …* ‘*Because we kept referring back to it,’.* ***(Participant 3).*** *‘I did get a very good booklet., So I knew exactly what was going on from day one****’ (Participant 2).*** *I read the booklet that they gave us originally, I went through that, and it did say that you can experience some pain and some numbness’* ***(Participant 3)***. *Was given a booklet before I had the operation and that …. gave me all the information I needed to know about, the actual operation, and what the after effects would be, how my recovery would take place‘, so I had a fairly good idea of what to expect’* ***(Participant 5)***. *‘I could keep referring back to the book’* ***(Participant 11).*** *‘As a patient I didn’t feel I needed to ask for any additional support, and I don’t know what would have happened had I have done. **(Participant 7).*** *‘I had a fairly good idea of what to expect… I’ve known a few people who’ve had surgery, and they said you need about three months to recover from it. So, I wasn't really going into the unknown’ (****Participant 5).*** *‘I was just bothered with up to the operation and what was going to happen’.****(Participant 6).***
Lack of contact/ continuity to reassure the patient. *‘I would have thought that probably I should have seen somebody after three weeks to put my mind at rest (****Participant 3).*** *Well, I just thought well, it [his leg pain] was a matter of course, but after two or three weeks I was thinking, is it right? Should I be getting in touch with somebody?****’*** ***(Participant 3).*** *‘Yes, it seems a long time from coming out of hospital with such a big operation and not seeing anyone for any reassurance until rehab’* ***(Participant 9).*** *I’m going to go 6 weeks basically before I see someone, I think it would have been an idea if I saw someone. Even if, I was told to go to the doctors after 3 weeks and make an appointment and then tell them if you’ve got any worries. Or for a district nurse to come round’* ***(Participant 10).*** *‘A swelling on the leg where they have opened up and there is nothing on it, nobody has told me to put one on.****’ (Participant 6).***

In the responses to the questions about person centred care the answers were very brief showing a lack of awareness and knowledge of person-centred care. (see Table 5)

**Table 5:** Person centred care.

How would you define person centred care?	In what ways were you involved in person centred care?
*‘Not really ever heard of it before’* **(***P****articipant 6).*** ‘*Somewhere you can go after an operation. A care centre or something’* ***(Participant 4).*** *‘Not sure’* ***(Participant 5).*** ‘*It’s probably they’re trying to improve the people experience in hospital and how they dealt with the staff and how the staff looked after them.’* ***(Participant 2).*** *I think whatever patient centered care is meant to be; it is not being delivered across the board.***’ *(Participant 1)***.	*I would still say a lot of it is about being able to do things yourself, not expecting a Nurse to come along and help you with something, you've got to get up and get yourself washed’* ***(Participant 6).*** ‘*I don’t say I was at the centre of it. I mean, I was reliant on them really. I mean, obviously they know what they’re doing so I’ve just got to go along with what they tell me really.*’ ***(Participant 4).*** *‘Not like physically or anything on the paperwork or anything that explained to me you are part of it, or anything like that’.* ***(Participant 2).*** *‘To a certain extent, yeah, I was involved, but I don't think I was at the heart of it.’* ***(Participant 5).***

## Discussion

A person-centred approach to discharge education is crucial in preparing patients both before their surgery and for their discharge home [27]. PCC means looking beyond the patient and understanding what matters to the person and this is fundamental in discharge planning. This study has provided a unique insight into the experience of the participants following a CABG. However, the study has revealed that some patients were ‘*not sure’* or ‘*never really heard of’* person centred care. Patients need to be educated about the benefits of PCC in managing their health and conditions by seeking support and planning care that is tailored to their individual needs [28]. PCC needs to place the individual at the centre of the care and decision making (NMC, 2024) [11].

The findings of our study align with those of Havana et al.’s (2023) [29] systematic review, which highlights that patients often have a limited understanding of PCC, feel excluded from the process, with few opportunities to voice their opinions, and may not be involved in decision-making and choices may be made on their behalf. In addition, Moore et al., (2017) [30], identified several barriers to implementing PCC, such as the fast pace of healthcare and the challenge of changing ingrained attitudes. Patients may not fully understand person-centred care due to poor communication from healthcare providers regarding how to participate in decision-making regarding their own care. More recently Kwarme & Petruka (2021) [20], identified that barriers to PCC and communication can be related to a shortage of nursing staff, high workload and shortage of time. These factors could lead to inconsistencies in how patients experience PCC, and a lack of uniformity may result in varying levels of engagement, making it challenging for patients to recognise PCC as a consistent, overarching principle in their healthcare experience.

Although person centred care is widely promoted in policy and clinical practice frameworks, there is an overarching perceived lack of patient understanding of PCC, it is acknowledged that most patients wish to be actively involved in managing their health and making decisions about their care [29]. However, the review of literature by Havana et al.’s, (2023) [29] identified that some patients prefer to take a more passive role, choosing to rely on healthcare professionals’ decisions rather than actively participating in the decision-making process. PCC was poorly understood by participants in our study, mirroring wider evidence that patients in hospital settings often struggle to articulate the concept [29]. PCC is predominantly defined by health care professionals and is seldom explained during routine clinical encounters, which limits patients’ ability to recognise or describe it in practice. Havana et al.’s (2023) review also highlighted the limited understanding of patient perspectives and revealed considerable variation in how PCC is experienced within hospital environments. Their findings emphasise the need to incorporate patient experiences in the development of PCC measurement tools and in the design and implementation of PCC initiatives. Furthermore, the review underscores the need for enhanced training for healthcare professionals to ensure consistent and meaningful delivery of person‑centred care**.**

Overall, our study demonstrated that patients valued the written information which was used during both the pre-and post-operative period. However, some participants expressed a need for more personalised education to prepare for discharge following CABG. Pre-operative clinic provides the opportunity to deliver patient education regarding the procedure and recovery period, yet some patients may feel unprepared due to a lack of clarity regarding the procedure or anxiety about the unknown. Martin et al.’s (2017) [31] [national, cross-sectional survey of 1917 patients (and caregivers) who had recent surgery, found that patients with access to more health information resources before and after surgery felt better prepared and had lower rates of 30-day readmission. Feeling prepared was positively correlated with the number of resources provided to patients by their surgical team. They also reported that the 30-day readmission was significantly lower among patients who felt prepared either before (7% prepared vs. 22% not prepared, *p* = < 0.001) or after surgery (9% prepared vs. 23% not prepared, *p* < 0.001). In our study, some patients commented on the lack of contact following discharge and felt less reassured, particularly related to wound care.

Previous research has highlighted the value of individualised education to ensure that advice is tailored to an individual's needs [16,21]. Our findings suggest that clear discharge planning and patient education is essential to ensure that patients feel prepared, confident, and ready to go home as well as empowered to seek further support and advice if needed.

The preparation and empowerment of patients is vital, especially given the theme identified in our study ‘not wanting to make a fuss / not seeking support’. As noted by Lateef (2020) [32], individuals often hesitate to ask for help due to concerns about burdening others and this reluctance can hinder access to necessary support. Despite patients not wanting to ‘make a fuss’, it is important to engage patients through reinforcement of education and information, to improve their health outcomes, knowledge and confidence [33]. It is also acknowledged that patient empowerment and engagement levels differ by race and ethnicity [33], and that patient expectations are based on a variety of factors such as health beliefs and levels of understanding. Our study was not designed or sufficiently diverse to examine how race and ethnicity can influence directly, but it is important to draw on this literature to acknowledge that engagement is shaped by multiple factors. A lack of engagement limits the opportunity for health care practitioners to provide reassurance and address misconceptions. Our findings suggest that whilst participants were fully prepared for surgery, patients did not seek post-operative advice. Patients seem to just want to get back to ‘normal’. This is also reported by Legler et al. (2019) [34] whose study of patient's post-acute coronary syndrome identified that 50% of the patients experienced gratitude to be alive and they wanted to ‘just get on with it’.

Globally, it is understood that health care provision should be evidence based and driven by practitioners who impart knowledge that increases patient confidence through empowerment [33]; [10]. Engaging patients in their health care decision making has significant benefits, including higher levels of satisfaction, increased knowledge and in some cases improved health outcomes. Hickmann et al. (2022) [35] emphasised that patients should be active partners in their care, enabling patients to become more empowered, leading to greater engagement. Engaging patients should allow for a more streamlined transition from hospital to home. Nevertheless, in our study some patients felt unprepared to go home and wanted to ‘stay an extra day’ despite being clinically fit for discharge, this may be due to patient anxiety about adapting to the home environment after surgery. Significantly, a study by Ji et al. (2022) [36] described that preoperative anxiety led to poorer post-operative outcomes. Equally, Krook et al’s (2020) [37] qualitative study explored the discharge process in Sweden and found that unplanned readmissions and lack of readiness of patients at discharge, were closely linked, recognizing a need to focus on information and communication at discharge. It is recognized that some Cardiologists may rush patients discharge to meet Key performance indicators (KPI’s)/ performance targets potentially ahead of patients being fully ready to be discharged home. (37). Our study supported this evidence, with one participant (4) stating that they “*felt quite vulnerable’* More recently, Chen et al (2026) [38] discuss same day discharge following PCI and identify that patients worry that carers might panic, and fear that their carer may be overly anxious and vigilant throughout the night if their PCI was undertaken by the femoral approach. In contrast, a recent study performed by Brlecic et al (2024) [39] identified that early discharge post CABG after isolated uncomplicated CABG had a significant association with reduced readmission up to 1 yr after CABG.

It is recognized that personalized approaches should enable patients to return home with confidence, limiting the incidence of unwarranted readmission and post-operative complications. PCC and personalized discussions can allow patients to develop realistic expectations during their care and can allow the HCP to fully understand the patient and their needs [28]. It is crucial that patients and their families are fully involved in the discharge planning [27]. Nevertheless, some participants in our study commented that they were not fully involved in person centred care and were reluctant to seek further support, perhaps due to a lack of awareness of what PCC entails or a lack of confidence in engaging in conversations or asking questions.

The participants relied on the booklet that was provided pre-operatively, this instilled a feeling of confidence, reducing the need to seek further information. Some participants relied on access to the internet to gain more information. However, it is recognised that information obtained through some non-professional sites may lack credibility and a strong evidence base [40]and patients should maintain contact with and communicate with HCPs to obtain the required information, yet the participants alluded to the fact that they didn't want to ‘meither’ or ‘make a fuss’ and therefore didn't contact the HCP’s. A move to increased digital healthcare [41] would help to empower patients with cardiac conditions, by providing reliable resources to educate them [42]. Telehealth also allows more accessible follow-ups and secure messaging platforms can facilitate better communication with patients addressing concerns and ensuring timely interventions [43]. Similarly, digitally enhanced cardiac rehabilitation structured education programmes such as, ‘Activate your heart’ (https://www.activateyourheart.org.uk/) allows patients to use the app on the move, providing an opportunity to ask health care professionals questions. Kenny et al (2023) [44] describe a study of 11 patients who participated in digital cardiac rehabilitation, they reported that this guided them toward recovery and improved their sense of empowerment but acknowledged that limited social interaction may be challenging for patients seeking social support.

Our findings showed that some participants felt there was a lack of contact from health care professionals on discharge from hospital. Some patients expected to see a clinician following discharge, but this didn’t always happen, patients had uncertainties about medication, pain relief and wound dressings, which required advice and support. However, as our findings have suggested, stoicism in patients post CABG has for some influenced their reluctance to seek advice post operation. Equally, staff may have assumed that the lack of questions was because patients felt confident with the discharge plans. A person-centred discharge conversation and individualized plan could have helped reduce this ambiguity and improved patient confidence. Early involvement from the family to help allay fears and fully prepare the patient may also have improved the patient's experience.

## Conclusion

Despite the high expectations and positive outlook about the benefits of the surgery, participants displayed issues with their discharge period due to being unprepared, not wanting to, or not knowing how to seek further support and purely relying on an information booklet. Preparing, empowering, and involving the patient in the discharge planning may have increased their confidence in the next steps of their recovery. Empowerment and preparation throughout the CABG journey from pre-operative to post surgery consultations is crucial to the patient's experience and ideally should become an integral part of services. Health care professionals need to recognize the stoicism of patients and provide individualized education to empower patients to have more confidence in managing their recovery. Individualized discharge education is key to ensuring that discharge information and education is tailored to the individual's needs. Individualised PCC will ensure that healthcare staff better understand what matters to the patient, rather than what is the matter *with* them.

### Implications for practice

Greater emphasis on PCC by health care professionals involved in the participants’ care may have resulted in a more rounded and individualized discharge process. In the discharge period following CABG the patient's lifestyle changes, and adaptations are required from both the patients and family members during the healing and recovery period [21]. Hence, PCC and individualized education may help to prepare the patient and the family / carers to be ready to face the potential challenges that they face [16]; [11].

### Limitations

Limitations of this study include the small number of participants, which although limited, falls within the recommended range for in-depth qualitative research [45]. The study emphasis was also pragmatic and the useful depth of patient stories and experiences rather than finding consensus. Additionally, all but one participant was white and all were English speaking, limiting ethnic diversity and cultural nuance. Participants were also recruited from a hospital in the North of England thereby potentially limiting generalisability across other geographical areas. In addition, although this study provides important insight into patients’ discharge experiences, data were collected in 2018–2019. The discussion draws on more recent policies and guidelines (2023–2024) to inform more recent practice, However, this temporal gap should be acknowledged, as changes in service models may influence the transferability of these findings to current practice.
