# Supplementary material for: Discharge after coronary artery bypass grafting: A qualitative study highlighting the need for individualised, person-centred care
Source: PLoS One. 2026 Jun 17;21(6):e0349948. doi: 10.1371/journal.pone.0349948 (PMC13274853; doi:10.1371/journal.pone.0349948)
Supplement: Attachment — Submitted filename: Peer Review Comments CABG PLOS ONE.docx [file pone.0349948.s001.docx]

Peer Review Comments

**Title of Manuscript:**

A qualitative exploration of patients’ discharge experience following Coronary Artery Bypass Graft – the need for more individualized person-centred care

Journal: PLOS ONE

------------------------------------------------------------

GENERAL COMMENTS

------------------------------------------------------------

This manuscript addresses an important and under-explored aspect of cardiac surgical care, namely patient experiences of discharge following CABG surgery. The focus on person-centred care (PCC) and discharge preparedness is timely and relevant to contemporary nursing and healthcare practice. The qualitative approach is appropriate, and the manuscript is generally well structured and clearly written. The use of constructivist grounded theory, COREQ guidance, and rich participant quotations enhances credibility. However, several methodological, analytical, and reporting issues need to be addressed before the manuscript can be considered for publication.

------------------------------------------------------------

MAJOR COMMENTS

------------------------------------------------------------

1. Methodological Clarity

Although the authors state that a constructivist grounded theory approach was adopted, the findings appear largely thematic and descriptive rather than theory-generating. The manuscript would benefit from clearer explanation of how grounded theory methods (e.g., memo writing, theoretical coding, core category development) were operationalised. If theory generation was not the primary aim, justification for the use of grounded theory over other qualitative approaches should be provided.

2. Sample Size and Information Power

The justification for the sample size using the concept of information power is appropriate but repeated across several sections of the manuscript. This repetition affects readability. Additionally, the distinction between thematic saturation and information power requires clearer explanation.

3. Timing of Data Collection

Data were collected between 2018 and 2019, whereas the discussion heavily references recent (2023–2024) policies and guidelines. The authors should explicitly acknowledge this temporal gap and discuss how changes in discharge practices and digital health delivery may influence the transferability of findings.

4. Reflexivity

Although researcher characteristics are described, reflexivity is limited. The authors should reflect on how their professional backgrounds as nurses and academics may have influenced data collection, participant responses, and interpretation of findings.

5. Integration of Person-Centred Care

While PCC is central to the study, participant understanding of the concept was limited. The discussion would benefit from deeper analytical engagement with why PCC was poorly understood by participants and how institutional or professional practices may contribute to this gap, rather than relying mainly on policy-level discussion.

------------------------------------------------------------

MINOR COMMENTS

------------------------------------------------------------

1. Abstract: Consider briefly interpreting the themes and adding a short statement on implications.

2. Title: Consider grammatical refinement to “patients’ discharge experience”.

3. Sample Description: Provide justification for the predominantly male sample and discuss its implications.

4. Tables: Table 4 is extensive; consider condensing or moving some quotations to supplementary material.

5. Language: Minor grammatical and typographical errors are present and require proofreading.

6. Ethics: Clarify consent procedures specifically for home interviews and audio recording.

------------------------------------------------------------

STRENGTHS OF THE MANUSCRIPT

------------------------------------------------------------

• Addresses a clinically important and under-researched phase of CABG care

• Rich qualitative data supported by participant quotations

• Use of COREQ checklist enhances transparency

• Clear implications for nursing and discharge planning practice

• Strong alignment with international person-centred care frameworks

------------------------------------------------------------

OVERALL RECOMMENDATION

------------------------------------------------------------

Major Revision

The manuscript has substantial merit and the potential to make a meaningful contribution to the literature. However, major revisions are required to strengthen methodological transparency, analytical depth, reflexivity, and contextual relevance.
