# Supplementary material for: Discharge after coronary artery bypass grafting: A qualitative study highlighting the need for individualised, person-centred care
Source: PLoS One. 2026 Jun 17;21(6):e0349948. doi: 10.1371/journal.pone.0349948 (PMC13274853; doi:10.1371/journal.pone.0349948)
Supplement: Attachment — Submitted filename: Response to reviewers PLOS one MR HIS.docx [file pone.0349948.s003.docx]

PONE-D-25-54340-

For the attention of:

Erfan Ghadirzadeh, MD
Academic Editor
PLOS One

Amended to :

Discharge After Coronary Artery Bypass Grafting: A Qualitative Study Highlighting the Need for Individualised, Person‑Centred Care

PLOS One

**Table 1**

**Items to be included when submitting revised manuscript**:

| **Item** | **Item** | **Completed and appendix number** |
| --- | --- | --- |
| **1** | - A letter that responds to each point raised by the academic editor and reviewer(s). You should upload this letter as a separate file labeled 'Response to Reviewers'. | **1** |
| **2** | - A marked-up copy of your manuscript that highlights changes made to the original version. You should upload this as a separate file labeled 'Revised Manuscript with Track Changes'. | **Yes** |
| **3** | - An unmarked version of your revised paper without tracked changes. You should upload this as a separate file labeled 'Manuscript'. | **Yes** |
| **4** | Please ensure that your manuscript meets PLOS ONE's style requirements, including those for file naming. The PLOS ONE style templates can be found at  [https://journals.plos.org/plosone/s/file?id=wjVg/PLOSOne_formatting_sample_main_body.pdf](https://eur01.safelinks.protection.outlook.com/?url=https%3A%2F%2Fjournals.plos.org%2Fplosone%2Fs%2Ffile%3Fid%3DwjVg%2FPLOSOne_formatting_sample_main_body.pdf&data=05%7C02%7CM.A.Rushton%40salford.ac.uk%7C7f6e5dcbc3424e9bbe8008de5cbe0853%7C65b52940f4b641bd833d3033ecbcf6e1%7C0%7C0%7C639050168438441823%7CUnknown%7CTWFpbGZsb3d8eyJFbXB0eU1hcGkiOnRydWUsIlYiOiIwLjAuMDAwMCIsIlAiOiJXaW4zMiIsIkFOIjoiTWFpbCIsIldUIjoyfQ%3D%3D%7C0%7C%7C%7C&sdata=cl82KOH7KEVhI0wOd5LNYIFJuiDPxSjAYBVrqmfjgro%3D&reserved=0) and  [https://journals.plos.org/plosone/s/file?id=ba62/PLOSOne_formatting_sample_title_authors_affiliations.pdf](https://eur01.safelinks.protection.outlook.com/?url=https%3A%2F%2Ftrack.editorialmanager.com%2FCL0%2Fhttps%3A%252F%252Fjournals.plos.org%252Fplosone%252Fs%252Ffile%253Fid%3Dba62%252FPLOSOne_formatting_sample_title_authors_affiliations.pdf%2F1%2F010f019bf84660e6-99c40bc6-cc2e-4e16-be6d-a7f93f56530c-000000%2FDns6MOm1ijhUZZBAR1tdwgh4n2s49EdEBuETpSiwGYs%3D247&data=05%7C02%7CM.A.Rushton%40salford.ac.uk%7C7f6e5dcbc3424e9bbe8008de5cbe0853%7C65b52940f4b641bd833d3033ecbcf6e1%7C0%7C0%7C639050168438481718%7CUnknown%7CTWFpbGZsb3d8eyJFbXB0eU1hcGkiOnRydWUsIlYiOiIwLjAuMDAwMCIsIlAiOiJXaW4zMiIsIkFOIjoiTWFpbCIsIldUIjoyfQ%3D%3D%7C0%7C%7C%7C&sdata=mcda9YCcpVXLaZWn5E2faWsK6jywXsypcxsmK8or0RE%3D&reserved=0) | **Yes** |
| **5** | Please amend your manuscript to include your abstract after the title page. | **Completed see page 2** |
| **6** | If the reviewer comments include a recommendation to cite specific previously published works, please review and evaluate these publications to determine whether they are relevant and should be cited. There is no requirement to cite these works unless the editor has indicated otherwise. | **OK** |
| **7** | To ensure your figures meet our technical requirements, please review our figure guidelines: [https://journals.plos.org/plosone/s/figures](https://eur01.safelinks.protection.outlook.com/?url=https%3A%2F%2Fjournals.plos.org%2Fplosone%2Fs%2Ffigures&data=05%7C02%7CM.A.Rushton%40salford.ac.uk%7C7f6e5dcbc3424e9bbe8008de5cbe0853%7C65b52940f4b641bd833d3033ecbcf6e1%7C0%7C0%7C639050168438633098%7CUnknown%7CTWFpbGZsb3d8eyJFbXB0eU1hcGkiOnRydWUsIlYiOiIwLjAuMDAwMCIsIlAiOiJXaW4zMiIsIkFOIjoiTWFpbCIsIldUIjoyfQ%3D%3D%7C0%7C%7C%7C&sdata=JVaTJMfqnyRedMAe3e%2FhEGdYLGR5yHWC68geHHGffuI%3D&reserved=0)  You may also use PLOS’s free figure tool, NAAS, to help you prepare publication quality figures: [https://journals.plos.org/plosone/s/figures#loc-tools-for-figure-preparation](https://eur01.safelinks.protection.outlook.com/?url=https%3A%2F%2Fjournals.plos.org%2Fplosone%2Fs%2Ffigures%23loc-tools-for-figure-preparation&data=05%7C02%7CM.A.Rushton%40salford.ac.uk%7C7f6e5dcbc3424e9bbe8008de5cbe0853%7C65b52940f4b641bd833d3033ecbcf6e1%7C0%7C0%7C639050168438663989%7CUnknown%7CTWFpbGZsb3d8eyJFbXB0eU1hcGkiOnRydWUsIlYiOiIwLjAuMDAwMCIsIlAiOiJXaW4zMiIsIkFOIjoiTWFpbCIsIldUIjoyfQ%3D%3D%7C0%7C%7C%7C&sdata=hMvWEOjFyTN%2FfZpPxOfNlYTP6odoz5wz%2BCsU9Joj2bA%3D&reserved=0).  NAAS will assess whether your figures meet our technical requirements by comparing each figure against our figure specifications. | **N/A** |

Melanie Rushton

University of Salford

A qualitative exploration of patients discharge experience following Coronary Artery Bypass Graft -the need for more individualized person- centred care.

Amended to :Discharge After Coronary Artery Bypass Grafting: A Qualitative Study Highlighting the Need for Individualised, Person‑Centred Care.

Manuscript ID PONE-D-25-54340

Date 20/03/26

Dear Erfan Ghadirzadeh (Editor PLOS one) and reviewers,

Thank you very much for the opportunity to revise and resubmit our manuscript ‘A qualitative exploration of patients discharge experience following Coronary Artery Bypass Graft -the need for more individualized person- centred care’. We have amended the title to ‘Discharge After Coronary Artery Bypass Grafting: A Qualitative Study Highlighting the Need for Individualised, Person‑Centred Care’.

We are very grateful for the feedback provided by the reviewers. We have carefully considered each comment and amended the manuscript accordingly. Below we provide a detailed point by point response. The table below identifies each of the reviewers’ comments and the responses include the line where the change appears in the manuscript.

We have prepared the following:

- 'Revised Manuscript with Track Changes’. A marked-up copy of our manuscript that highlights changes made to the original version.
- 'Manuscript' An unmarked version of our revised paper without tracked changes.

We trust that the revisions address the comments made by the reviewers and we sincerely appreciate the time and expertise invested in evaluating our work. We hope the revised manuscript will be suitable for publication in PLOS one.

Thank you for your consideration and we look forward to hearing from you.

Kind regards

Melanie Rushton

University of Salford

m.a.rushton@salford.ac.uk

**Reviewer comments and action**

**Reviewer 1**

| **Comments** | **Response (line stated refer to the revised manuscript with track changes** |
| --- | --- |
| Methodological Clarity Although the authors state that a constructivist grounded theory approach was adopted, the findings appear largely thematic and descriptive rather than theory-generating. The manuscript would benefit from clearer explanation of how grounded theory methods (e.g., memo writing, theoretical coding, core category development) were operationalised. If theory generation was not the primary aim, justification for the use of grounded theory over other qualitative approaches should be provided. | **Added to line 170 :**  The primary objective of this study was not to develop a formal theory, but the adapted constructivist grounded theory approach was selected because of its methodological suitability for examining complex phenomena such as discharge experience following CABG as it allows a systematic and iterative analytical structure, through constant comparison, layered coding and memo writing. |
| Sample Size and Information Power The justification for the sample size using the concept of information power is appropriate but repeated across several sections of the manuscript. This repetition affects readability. Additionally, the distinction between thematic saturation and information power requires clearer explanation. | **Line 183 Deleted:** Thematic saturation was observed early in the analysis, indicating that the data sufficiently addressed the research questions  **Added in line 227** : In this study, sample adequacy was evaluated using information power rather than relying solely on thematic saturation. Information power considers the relevance, specificity and depth of data in relation to the study aim. The focused research question and richness of participant narratives ensured that information power was sufficient without necessitating complete thematic redundancy. |
| Timing of Data Collection Data were collected between 2018 and 2019, whereas the discussion heavily references recent (2023–2024) policies and guidelines. The authors should explicitly acknowledge this temporal gap and discuss how changes in discharge practices and digital health delivery may influence the transferability of findings. | **Line 178 added in re data collection**  As data were collected between 2018 and 2019, readers should consider the potential temporal gap when interpreting the findings from the study, as cardiac services and discharge practices have evolved in subsequent years inclusive of digital health practices.  **Line 503 added in**  In addition, although this study provides important insight into patients’ discharge experiences, data were collected 2018–2019. The discussion draws on more recent policies and guidelines (2023–2024) to inform more recent practice. However, this temporal gap should be acknowledged, as changes in service models may influence the transferability of these findings to current practice. |
| Reflexivity Although researcher characteristics are described, reflexivity is limited. The authors should reflect on how their professional backgrounds as nurses and academics may have influenced data collection, participant responses, and interpretation of findings. | **Line 236 added into Methods section:**  Data were collected by experienced nurse academic researchers MR and MH both white females, the first, a cardiac nurse with a MA in Education Research and the second a registered nurse with a PhD, both employed as academics which, may have influenced how interviews were conducted and interpreted. |
| Integration of Person-Centred Care While PCC is central to the study, participant understanding of the concept was limited. The discussion would benefit from deeper analytical engagement with why PCC was poorly understood by participants and how institutional or professional practices may contribute to this gap, rather than relying mainly on policy-level discussion. | **Line 368 added in :**  PCC was poorly understood by participants in our study, mirroring wider evidence that patients in hospital settings often struggle to articulate the concept (Havana et al (2023). PCC is predominantly defined by health care professionals and is seldom explained during routine clinical encounters, which limits patients’ ability to recognise or describe it in practice. Havana et al.’s (2023) review also highlighted the limited understanding of patient perspectives and revealed considerable variation in how PCC is experienced within hospital environments. Their findings emphasise the need to incorporate patient experiences in the development of PCC measurement tools and in the design and implementation of PCC initiatives. Furthermore, the review underscores the need for enhanced training for healthcare professionals to ensure consistent and meaningful delivery of person‑centred care**.** |
| Abstract: Consider briefly interpreting the themes and adding a short statement on implications. | **Line 61** **added the following :**  The participants’ reluctance to seek support could reflect a pattern of self-management shaped by uncertainty, low confidence or a desire not to burden healthcare professionals. Yet, many felt unprepared for discharge perhaps highlighting gaps in communication and discharge planning. There also seems a reliance on written materials such as the information booklet. Limited post- discharge contact, created further anxiety, where patients were unsure where to turn for advice.  These findings point to the need for clearer, more person‑centred discharge processes. Better communication, improved pre‑discharge education, and clear pathways for follow‑up support could strengthen patient confidence and safety. Although booklets are helpful, they should supplement and not replace ongoing contact and personalised guidance. Ensuring continuity of care and enabling patients to seek help without hesitation is essential for effective person‑centred discharge practice. |
| Title: Consider grammatical refinement to “patients’ discharge experience”. | **Amended to :** Discharge After Coronary Artery Bypass Grafting: A Qualitative Study Highlighting the Need for Individualised, Person‑Centred Care |
| Sample Description: Provide justification for the predominantly male sample and discuss its implications. | **Added into line 298** The sample was predominantly male as the pool of eligible participants reflected the clinical population undergoing CABG surgery. Epidemiologically, men are significantly more likely to develop coronary artery disease (CAD) at a younger age requiring interventions. The majority of patients available for recruitment at the time were male, therefore the gender imbalance was a natural characteristic of the sampling rather than a recruitment bias. |
| Tables: Table 4 is extensive; consider condensing or moving some quotations to supplementary material. | **please see line 323 (Table 4)** We appreciate the reviewer’s suggestion and have already condensed parts of the section where possible. I have amended the format of the table to take up less space The quotations included are essential for demonstrating transparency and ensuring that each theme is sufficiently supported by primary data. |
| Language: Minor grammatical and typographical errors are present and require proofreading. | Microsoft word editor used to amend grammatical and typographical errors  The word centred used in text and centered in some references.  Some spellings are highlighted but the speeling presented is the English version  Participant quotations and references include some grammatical errors which can’t be amended |
| Ethics: Clarify consent procedures specifically for home interviews and audio recording. | **Line 242- added:**  Before conducting home-based interviews, all participants had an information sheet outlining the study purpose, procedures, voluntary participation and the right to withdraw. At the scheduled home visit the researcher verbally reiterated the information, answered the participants questions and confirmed that the participant had fully understood the study. Written informed consent was obtained before the interview began, which included consent to the audio recording of the interview, Participants were reminded that they could withdraw or skip questions without providing a reason. The Informed written consent was securely stored in accordance with institutional data protection policies. |
| major revisions are required to strengthen methodological transparency, analytical depth, reflexivity, and contextual relevance. | I have revised the areas identified and are detailed above |

**Reviewer 2**

| **Comments** | **Response** |
| --- | --- |
| On p.4, in line 101, the correct phrase should be "Registered Nurse Standards for Practice in Australia" rather than "the Australian Nursing Professional Standards". This phrase, "the Australian Professional Standards," appears misleading because it suggests the standards apply to both enrolled and registered nurses in Australia. It is imperative to note that there is a separate standards document for enrolled nurses in Australia. It is called Enrolled Nurse Standards for Practice. Hence, the need to use the "Registered Nurses Standards for Practice" or simply use "Australian professional nursing standards". Please pay attention to my lack of proper nouns and adjectives in the phrase; doing so would encompass both the enrolled and registered nurses' standards of practice. Although Byrne et al. (2020) used the phrase in their paper, they did so to distinguish the standards from other global nursing standards. | **Thank you I have amended this as advised on P 4 in line 142** |
| In lines 99-100, where you stated "In their Australian research …", you need to cite other works from Byrnes et al. or change the word "research" to "review". | **Thankyou- I have changed the word to review (lines 140 )** |
| **Methods** -What rationale did you use when you chose 4-6 weeks post-CABG? The reasoning would help readers. Then, in line 143, you stated you chose two to five weeks post-discharge to help participants recall events. | **-I have reworded this to make the rationale clearer (lines 203- 210)**  The interview timeline (two to five weeks post discharge) was chosen to maximise accurate recall while allowing initial recovery and preventing memory decay. The 2-5 week period was sufficiently recent to aide memory recall whilst providing time for the participant to recover from major surgery. Additionally, cardiac rehabilitation commonly begins at 6 weeks and therefore interviewing before this time reduces the prospect of the participants experiences being influenced. The timing helps maintain internal validity, ensuring that insights relate genuinely to the early recovery phase rather than to formal rehabilitation content. |
| **Study participants** -On discharge, it is reasonable for patients to feel that any nursing contact could be part of their discharge plan. I acknowledge that the participants' information sheet and consent form would spell everything out, but there would be a feeling that their discharge plan could be undermined if they declined to participate in your research. How did you manage power imbalances when you approached potential research participants? | **- added the following to lines 194- 200:**  We recognized that the recruitment process involved power imbalances, as patients were approached by a member of the pre operative assessment clinic team in a pre-surgical context. This may have influenced patients’ perceived ability to decline participation. To mitigate this voluntariness was emphasised, we provided time for them to decide. The researchers who contacted them were not involved in their care, it was made clear that declining would not affect treatment  ‘ |
| **-Data collection.** The word 'data' is the plural form of 'datum' and should have the correct verb and other grammatical mechanics. | I have checked through the document and where we refer to data this is pleural as referring to multiple |
| You stated you obtained consent from the participants, but it is not clear whether some participants did not consent prior to data collection. One thing is to provide information to participants, but another is to give or withhold consent. | **I have added the following into line 189**  All patients who fit the inclusion criteria were asked in the preoperative assessment clinic clinic if they wanted to be part of the research with a full explanation and consent obtained for the researchers to contact on discharge. However, those patients who declined consent at any part of the process were not included in the study, this did not affect their treatment plan. |
| In line 152, did you mean information power led to the emergent themes? | **Yes, I have amended this (now line 217 )** |
| Discussion. -You mentioned race and ethnicity affect how they engage with their discharge information, but it is not clear how your findings integrated this study. | **I have added into line 400** Our study was not designed or sufficiently diverse to examine how race and ethnicity can influence directly, but it is important to draw on this literature to acknowledge that engagement is shaped by multiple factors. |
| It is well established that some cardiologists rush patients' discharges to meet KPIs even though these patients may not be "fit for discharge". Please can you relate your findings to this area of concern? | **Line 423 added in the following**- It is recognized that some Cardiologists may rush patient discharge to meet health care key performance indicators KPI’s/performance targets potential ahead of patients being fully ready to be discharged home (Krook et al 2020). Our study supported this evidence, with one participant (4) stating that they ‘*‘felt quite vulnerable’*. More recently Chen et al (2026)[38] discuss same day discharge following PCI and identify that patients worry that carers might panic, and fear that their carer may be overly anxious and vigilant throughout the night if their PCI was undertaken by the femoral approach. In contrast, a recent study performed by Brlecic et al (2024) [39] identified that early discharge post CABG after isolated uncomplicated CABG had a significant association with reduced readmission up to 1 yr after CABG. |
| **Implications for practice** **Limitations** You demonstrated broad perspectives of your findings when you mentioned the lack of cultural diversity in your research. | **Yes this is on line 494** Additionally, all but one participant was white and all were English speaking, limiting ethnic diversity and cultural nuance |
