# Supplementary material for: Discharge after coronary artery bypass grafting: A qualitative study highlighting the need for individualised, person-centred care
Source: PLoS One. 2026 Jun 17;21(6):e0349948. doi: 10.1371/journal.pone.0349948 (PMC13274853; doi:10.1371/journal.pone.0349948)
Supplement: Peer review history

# Peer review: Decision Letter 0

**DOI:** 10.1371/journal.pone.0349948.r001

**Authors:** Erfan Ghadirzadeh

25 Jan 2026

PONE-D-25-54340

A qualitative exploration of patients discharge experience following Coronary Artery Bypass Graft -the need for more individualized person- centred care.

PLOS One

Dear Dr. Rushton,

Thank you for submitting your manuscript to PLOS ONE. After careful consideration, we feel that it has merit but does not fully meet PLOS ONE’s publication criteria as it currently stands. Therefore, we invite you to submit a revised version of the manuscript that addresses the points raised during the review process.

Please submit your revised manuscript by Mar 11 2026 11:59PM. If you will need more time than this to complete your revisions, please reply to this message or contact the journal office at plosone@plos.org. When you're ready to submit your revision, log on to https://www.editorialmanager.com/pone/ and select the 'Submissions Needing Revision' folder to locate your manuscript file.

Please include the following items when submitting your revised manuscript:

- A letter that responds to each point raised by the academic editor and reviewer(s). You should upload this letter as a separate file labeled 'Response to Reviewers'.
- A marked-up copy of your manuscript that highlights changes made to the original version. You should upload this as a separate file labeled 'Revised Manuscript with Track Changes'.
- An unmarked version of your revised paper without tracked changes. You should upload this as a separate file labeled 'Manuscript'.

If you would like to make changes to your financial disclosure, please include your updated statement in your cover letter. Guidelines for resubmitting your figure files are available below the reviewer comments at the end of this letter.

If applicable, we recommend that you deposit your laboratory protocols in protocols.io to enhance the reproducibility of your results. Protocols.io assigns your protocol its own identifier (DOI) so that it can be cited independently in the future. For instructions see: https://journals.plos.org/plosone/s/submission-guidelines#loc-laboratory-protocols. Additionally, PLOS ONE offers an option for publishing peer-reviewed Lab Protocol articles, which describe protocols hosted on protocols.io. Read more information on sharing protocols at https://plos.org/protocols?utm_medium=editorial-email&utm_source=authorletters&utm_campaign=protocols.

We look forward to receiving your revised manuscript.

Kind regards,

Erfan Ghadirzadeh, MD

Academic Editor

PLOS One

Journal Requirements:

When submitting your revision, we need you to address these additional requirements.

1. Please ensure that your manuscript meets PLOS ONE's style requirements, including those for file naming. The PLOS ONE style templates can be found at

https://journals.plos.org/plosone/s/file?id=wjVg/PLOSOne_formatting_sample_main_body.pdf and

https://journals.plos.org/plosone/s/file?id=ba62/PLOSOne_formatting_sample_title_authors_affiliations.pdf

2. Please amend your manuscript to include your abstract after the title page.

3. If the reviewer comments include a recommendation to cite specific previously published works, please review and evaluate these publications to determine whether they are relevant and should be cited. There is no requirement to cite these works unless the editor has indicated otherwise.

[Note: HTML markup is below. Please do not edit.]

Reviewers' comments:

Reviewer's Responses to Questions

**Comments to the Author**

1. Is the manuscript technically sound, and do the data support the conclusions?

The manuscript must describe a technically sound piece of scientific research with data that supports the conclusions. Experiments must have been conducted rigorously, with appropriate controls, replication, and sample sizes. The conclusions must be drawn appropriately based on the data presented.

Reviewer #1: Partly

Reviewer #2: Yes

**********

2. Has the statistical analysis been performed appropriately and rigorously?

Reviewer #1: Yes

Reviewer #2: N/A

**********

3. Have the authors made all data underlying the findings in their manuscript fully available?

The PLOS Data policy requires authors to make all data underlying the findings described in their manuscript fully available without restriction, with rare exception (please refer to the Data Availability Statement in the manuscript PDF file). The data should be provided as part of the manuscript or its supporting information, or deposited to a public repository. For example, in addition to summary statistics, the data points behind means, medians and variance measures should be available. If there are restrictions on publicly sharing data—e.g. participant privacy or use of data from a third party—those must be specified.

Reviewer #1: Yes

Reviewer #2: No

**********

4. Is the manuscript presented in an intelligible fashion and written in standard English?

PLOS ONE does not copyedit accepted manuscripts, so the language in submitted articles must be clear, correct, and unambiguous. Any typographical or grammatical errors should be corrected at revision, so please note any specific errors here.

Reviewer #1: Yes

Reviewer #2: No

**********

5. Review Comments to the Author

Please use the space provided to explain your answers to the questions above. You may also include additional comments for the author, including concerns about dual publication, research ethics, or publication ethics. (Please upload your review as an attachment if it exceeds 20,000 characters)

Reviewer #1: GENERAL COMMENTS:

This manuscript addresses an important and under-explored aspect of cardiac surgical care, namely patient experiences of discharge following CABG surgery. The focus on person-centred care (PCC) and discharge preparedness is timely and relevant to contemporary nursing and healthcare practice. The qualitative approach is appropriate, and the manuscript is generally well structured and clearly written. The use of constructivist grounded theory, COREQ guidance, and rich participant quotations enhances credibility. However, several methodological, analytical, and reporting issues need to be addressed before the manuscript can be considered for publication.

MAJOR COMMENTS:

1. Methodological Clarity

Although the authors state that a constructivist grounded theory approach was adopted, the findings appear largely thematic and descriptive rather than theory-generating. The manuscript would benefit from clearer explanation of how grounded theory methods (e.g., memo writing, theoretical coding, core category development) were operationalised. If theory generation was not the primary aim, justification for the use of grounded theory over other qualitative approaches should be provided.

2. Sample Size and Information Power

The justification for the sample size using the concept of information power is appropriate but repeated across several sections of the manuscript. This repetition affects readability. Additionally, the distinction between thematic saturation and information power requires clearer explanation.

3. Timing of Data Collection

Data were collected between 2018 and 2019, whereas the discussion heavily references recent (2023–2024) policies and guidelines. The authors should explicitly acknowledge this temporal gap and discuss how changes in discharge practices and digital health delivery may influence the transferability of findings.

4. Reflexivity

Although researcher characteristics are described, reflexivity is limited. The authors should reflect on how their professional backgrounds as nurses and academics may have influenced data collection, participant responses, and interpretation of findings.

5. Integration of Person-Centred Care

While PCC is central to the study, participant understanding of the concept was limited. The discussion would benefit from deeper analytical engagement with why PCC was poorly understood by participants and how institutional or professional practices may contribute to this gap, rather than relying mainly on policy-level discussion.

MINOR COMMENTS:

1. Abstract: Consider briefly interpreting the themes and adding a short statement on implications.

2. Title: Consider grammatical refinement to “patients’ discharge experience”.

3. Sample Description: Provide justification for the predominantly male sample and discuss its implications.

4. Tables: Table 4 is extensive; consider condensing or moving some quotations to supplementary material.

5. Language: Minor grammatical and typographical errors are present and require proofreading.

6. Ethics: Clarify consent procedures specifically for home interviews and audio recording.

STRENGTHS OF THE MANUSCRIPT

• Addresses a clinically important and under-researched phase of CABG care

• Rich qualitative data supported by participant quotations

• Use of COREQ checklist enhances transparency

• Clear implications for nursing and discharge planning practice

• Strong alignment with international person-centred care frameworks

OVERALL RECOMMENDATION:

Major Revision

The manuscript has substantial merit and the potential to make a meaningful contribution to the literature. However, major revisions are required to strengthen methodological transparency, analytical depth, reflexivity, and contextual relevance.

Reviewer #2: Introduction

On p.4, in line 101, the correct phrase should be "Registered Nurse Standards for Practice in Australia" rather than "the Australian Nursing Professional Standards". This phrase, "the Australian Professional Standards," appears misleading because it suggests the standards apply to both enrolled and registered nurses in Australia. It is imperative to note that there is a separate standards document for enrolled nurses in Australia. It is called Enrolled Nurse Standards for Practice. Hence, the need to use the "Registered Nurses Standards for Practice" or simply use "Australian professional nursing standards". Please pay attention to my lack of proper nouns and adjectives in the phrase; doing so would encompass both the enrolled and registered nurses' standards of practice. Although Byrne et al. (2020) used the phrase in their paper, they did so to distinguish the standards from other global nursing standards.

In lines 99-100, where you stated "In their Australian research …", you need to cite other works from Byrnes et al. or change the word "research" to "review".

Methods

What rationale did you use when you chose 4-6 weeks post-CABG? The reasoning would help readers. Then, in line 143, you stated you chose two to five weeks post-discharge to help participants recall events.

Study participants

On discharge, it is reasonable for patients to feel that any nursing contact could be part of their discharge plan. I acknowledge that the participants' information sheet and consent form would spell everything out, but there would be a feeling that their discharge plan could be undermined if they declined to participate in your research. How did you manage power imbalances when you approached potential research participants?

Data collection.

The word 'data' is the plural form of 'datum' and should have the correct verb and other grammatical mechanics.

You stated you obtained consent from the participants, but it is not clear whether some participants did not consent prior to data collection. One thing is to provide information to participants, but another is to give or withhold consent.

It is great to see that you avoided the phrase "data saturation" and instead used "information power," which is more appropriate for a qualitative study. Especially in grounded theory, where you must generate a theory from the data (participants' interpretations of their experiences), because themes are not hidden within the data.

In line 152, did you mean information power led to the emergent themes?

Discussion.

You mentioned race and ethnicity affect how they engage with their discharge information, but it is not clear how your findings integrated this study.

It is well established that some cardiologists rush patients' discharges to meet KPIs even though these patients may not be "fit for discharge". Please can you relate your findings to this area of concern?

Implications for practice

Limitations

You demonstrated broad perspectives of your findings when you mentioned the lack of cultural diversity in your research.

**********

6. PLOS authors have the option to publish the peer review history of their article (what does this mean?). If published, this will include your full peer review and any attached files.

If you choose “no”, your identity will remain anonymous but your review may still be made public.

**Do you want your identity to be public for this peer review?**   For information about this choice, including consent withdrawal, please see our Privacy Policy.

Reviewer #1: **Yes:**  SARANYA R

Reviewer #2: **Yes:**  Adeniyi Olanrewaju Adeleye

**********

[NOTE: If reviewer comments were submitted as an attachment file, they will be attached to this email and accessible via the submission site. Please log into your account, locate the manuscript record, and check for the action link "View Attachments". If this link does not appear, there are no attachment files.]

To ensure your figures meet our technical requirements, please review our figure guidelines: https://journals.plos.org/plosone/s/figures

You may also use PLOS’s free figure tool, NAAS, to help you prepare publication quality figures: https://journals.plos.org/plosone/s/figures#loc-tools-for-figure-preparation.

NAAS will assess whether your figures meet our technical requirements by comparing each figure against our figure specifications.

Attachment Submitted filename: Peer Review Comments CABG PLOS ONE.docx

# Author comment: Author response to Decision Letter 1

**DOI:** 10.1371/journal.pone.0349948.r002

20 Mar 2026

Reviewer comments and action

Reviewer 1

1-Methodological Clarity

Although the authors state that a constructivist grounded theory approach was adopted, the findings appear largely thematic and descriptive rather than theory-generating. The manuscript would benefit from clearer explanation of how grounded theory methods (e.g., memo writing, theoretical coding, core category development) were operationalised. If theory generation was not the primary aim, justification for the use of grounded theory over other qualitative approaches should be provided.

I have added the following to line 170 :

The primary objective of this study was not to develop a formal theory, but the adapted constructivist grounded theory approach was selected because of its methodological suitability for examining complex phenomena such as discharge experience following CABG as it allows a systematic and iterative analytical structure, through constant comparison, layered coding and memo writing.

2- Sample Size and Information Power

The justification for the sample size using the concept of information power is appropriate but repeated across several sections of the manuscript. This repetition affects readability. Additionally, the distinction between thematic saturation and

information power requires clearer explanation.

Line 183 Deleted: Thematic saturation was observed early in the analysis, indicating that the data sufficiently addressed the research questions

Added in line 227 : In this study, sample adequacy was evaluated using information power rather than relying solely on thematic saturation. Information power considers the relevance, specificity and depth of data in relation to the study aim. The focused research question and richness of participant narratives ensured that information power was sufficient without necessitating complete thematic redundancy.

3- Timing of Data Collection

Data were collected between 2018 and 2019, whereas the discussion heavily references recent (2023–2024) policies and guidelines. The authors should explicitly acknowledge this temporal gap and discuss how changes in discharge practices and digital health delivery may influence the transferability of findings.

Line 178 added in re data collection

As data were collected between 2018 and 2019, readers should consider the potential temporal gap when interpreting the findings from the study, as cardiac services and discharge practices have evolved in subsequent years inclusive of digital health practices.

Line 503 added in

In addition, although this study provides important insight into patients’ discharge experiences, data were collected 2018–2019. The discussion draws on more recent policies and guidelines (2023–2024) to inform more recent practice. However, this temporal gap should be acknowledged, as changes in service models may influence the transferability of these findings to current practice.

4- Reflexivity

Although researcher characteristics are described, reflexivity is limited. The authors should reflect on how their professional backgrounds as nurses and academics may have influenced data collection, participant responses, and interpretation of findings.

Line 236 added into Methods section:

Data were collected by experienced nurse academic researchers MR and MH both white females, the first, a cardiac nurse with a MA in Education Research and the second a registered nurse with a PhD, both employed as academics which, may have influenced how interviews were conducted and interpreted.

5- Integration of Person-Centred Care

While PCC is central to the study, participant understanding of the concept was limited. The discussion would benefit from deeper analytical engagement with why PCC was poorly understood by participants and how institutional or professional practices may contribute to this gap, rather than relying mainly on policy-level discussion.

Line 368 added in :

PCC was poorly understood by participants in our study, mirroring wider evidence that patients in hospital settings often struggle to articulate the concept (Havana et al (2023). PCC is predominantly defined by health care professionals and is seldom explained during routine clinical encounters, which limits patients’ ability to recognise or describe it in practice. Havana et al.’s (2023) review also highlighted the limited understanding of patient perspectives and revealed considerable variation in how PCC is experienced within hospital environments. Their findings emphasise the need to incorporate patient experiences in the development of PCC measurement tools and in the design and implementation of PCC initiatives. Furthermore, the review underscores the need for enhanced training for healthcare professionals to ensure consistent and meaningful delivery of person centred care.

6- Abstract: Consider briefly interpreting the themes and adding a short statement on implications.

Line 61 added the following :

The participants’ reluctance to seek support could reflect a pattern of self-management shaped by uncertainty, low confidence or a desire not to burden healthcare professionals. Yet, many felt unprepared for discharge perhaps highlighting gaps in communication and discharge planning. There also seems a reliance on written materials such as the information booklet. Limited post- discharge contact, created further anxiety, where patients were unsure where to turn for advice.

These findings point to the need for clearer, more person centred discharge processes. Better communication, improved pre discharge education, and clear pathways for follow up support could strengthen patient confidence and safety. Although booklets are helpful, they should supplement and not replace ongoing contact and personalised guidance. Ensuring continuity of care and enabling patients to seek help without hesitation is essential for effective person centred discharge practice.

7- Title: Consider grammatical refinement to “patients’ discharge experience”.

Amended to : Discharge After Coronary Artery Bypass Grafting: A Qualitative Study Highlighting the Need for Individualised, Person Centred Care

8- Sample Description: Provide justification for the predominantly male sample and discuss its implications.

Added into line 298 The sample was predominantly male as the pool of eligible participants reflected the clinical population undergoing CABG surgery. Epidemiologically, men are significantly more likely to develop coronary artery disease (CAD) at a younger age requiring interventions. The majority of patients available for recruitment at the time were male, therefore the gender imbalance was a natural characteristic of the sampling rather than a recruitment bias.

9- Tables: Table 4 is extensive; consider condensing or moving some quotations to supplementary material.

please see line 323 (Table 4) We appreciate the reviewer’s suggestion and have already condensed parts of the section where possible. I have amended the format of the table to take up less space The quotations included are essential for demonstrating transparency and ensuring that each theme is sufficiently supported by primary data.

10- Language: Minor grammatical and typographical errors are present and require proofreading.

Microsoft word editor used to amend grammatical and typographical errors

The word centred used in text and centered in some references.

Some spellings are highlighted but the speeling presented is the English version

Participant quotations and references include some grammatical errors which can’t be amended

11 Ethics: Clarify consent procedures specifically for home interviews and audio recording.

Line 242- added:

Before conducting home-based interviews, all participants had an information sheet outlining the study purpose, procedures, voluntary participation and the right to withdraw. At the scheduled home visit the researcher verbally reiterated the information, answered the participants questions and confirmed that the participant had fully understood the study. Written informed consent was obtained before the interview began, which included consent to the audio recording of the interview, Participants were reminded that they could withdraw or skip questions without providing a reason. The Informed written consent was securely stored in accordance with institutional data protection policies.

12- major revisions are required to strengthen methodological transparency, analytical depth, reflexivity, and contextual relevance.

I have revised the areas identified and are detailed above

Reviewer 2

1- On p.4, in line 101, the correct phrase should be "Registered Nurse Standards for Practice in Australia" rather than "the Australian Nursing Professional Standards". This phrase, "the Australian Professional Standards," appears misleading because it suggests the standards apply to both enrolled and registered nurses in Australia. It is imperative to note that there is a separate standards document for enrolled nurses in Australia. It is called Enrolled Nurse Standards for Practice. Hence, the need to use the "Registered Nurses Standards for Practice" or simply use "Australian professional nursing standards". Please pay attention to my lack of proper nouns and adjectives in the phrase; doing so would encompass both the enrolled and registered nurses' standards of practice. Although Byrne et al. (2020) used the phrase in their paper, they did so to distinguish the standards from other global nursing standards.

Thank you I have amended this as advised on P 4 in line 142

2- In lines 99-100, where you stated "In their Australian research …", you need to cite other works from Byrnes et al. or change the word "research" to "review".

Thankyou- I have changed the word to review (lines 140 )

3- Methods

-What rationale did you use when you chose 4-6 weeks post-CABG? The reasoning would help readers. Then, in line 143, you stated you chose two to five weeks post-discharge to help participants recall events.

-I have reworded this to make the rationale clearer (lines 203- 210)

The interview timeline (two to five weeks post discharge) was chosen to maximise accurate recall while allowing initial recovery and preventing memory decay. The 2-5 week period was sufficiently recent to aide memory recall whilst providing time for the participant to recover from major surgery. Additionally, cardiac rehabilitation commonly begins at 6 weeks and therefore interviewing before this time reduces the prospect of the participants experiences being influenced. The timing helps maintain internal validity, ensuring that insights relate genuinely to the early recovery phase rather than to formal rehabilitation content.

4- Study participants

-On discharge, it is reasonable for patients to feel that any nursing contact could be part of their discharge plan. I acknowledge that the participants' information sheet and consent form would spell everything out, but there would be a feeling that their discharge plan could be undermined if they declined to participate in your research. How did you manage power imbalances when you approached potential research participants?

- added the following to lines 194- 200:

We recognized that the recruitment process involved power imbalances, as patients were approached by a member of the pre operative assessment clinic team in a pre-surgical context. This may have influenced patients’ perceived ability to decline participation. To mitigate this voluntariness was emphasised, we provided time for them to decide. The researchers who contacted them were not involved in their care, it was made clear that declining would not affect treatment

5- Data collection.

The word 'data' is the plural form of 'datum' and should have the correct verb and other grammatical mechanics

I have checked through the document and where we refer to data this is pleural as referring to multiple

6- You stated you obtained consent from the participants, but it is not clear whether some participants did not consent prior to data collection. One thing is to provide information to participants, but another is to give or withhold consent.

I have added the following into line 189

All patients who fit the inclusion criteria were asked in the preoperative assessment clinic clinic if they wanted to be part of the research with a full explanation and consent obtained for the researchers to contact on discharge. However, those patients who declined consent at any part of the process were not included in the study, this did not affect their treatment plan.

7- In line 152, did you mean information power led to the emergent themes?

Yes, I have amended this (now line 217 )

8- Discussion.

-You mentioned race and ethnicity affect how they engage with their discharge information, but it is not clear how your findings integrated this study.

I have added into line 400 Our study was not designed or sufficiently diverse to examine how race and ethnicity can influence directly, but it is important to draw on this literature to acknowledge that engagement is shaped by multiple factors.

9- It is well established that some cardiologists rush patients' discharges to meet KPIs even though these patients may not be "fit for discharge". Please can you relate your findings to this area of concern?

Line 423 added in the following- It is recognized that some Cardiologists may rush patient discharge to meet health care key performance indicators KPI’s/performance targets potential ahead of patients being fully ready to be discharged home (Krook et al 2020). Our study supported this evidence, with one participant (4) stating that they ‘‘felt quite vulnerable’. More recently Chen et al (2026)[38] discuss same day discharge following PCI and identify that patients worry that carers might panic, and fear that their carer may be overly anxious and vigilant throughout the night if their PCI was undertaken by the femoral approach. In contrast, a recent study performed by Brlecic et al (2024) [39] identified that early discharge post CABG after isolated uncomplicated CABG had a significant association with reduced readmission up to 1 yr after CABG

10- Implications for practice

Limitations

You demonstrated broad perspectives of your findings when you mentioned the lack of cultural diversity in your research.

Yes this is on line 494 Additionally, all but one participant was white and all were English speaking, limiting ethnic diversity and cultural nuance

Attachment Submitted filename: Response to reviewers PLOS one MR HIS.docx

# Peer review: Decision Letter 1

**DOI:** 10.1371/journal.pone.0349948.r003

**Authors:** Erfan Ghadirzadeh

8 May 2026

Discharge After Coronary Artery Bypass Grafting: A Qualitative Study Highlighting the Need for Individualised, Person‑Centred Care.

PONE-D-25-54340R1

Dear Dr. Rushton,

We’re pleased to inform you that your manuscript has been judged scientifically suitable for publication and will be formally accepted for publication once it meets all outstanding technical requirements.

Within one week, you’ll receive an e-mail detailing the required amendments. When these have been addressed, you’ll receive a formal acceptance letter and your manuscript will be scheduled for publication.

An invoice will be generated when your article is formally accepted. Please note, if your institution has a publishing partnership with PLOS and your article meets the relevant criteria, all or part of your publication costs will be covered. Please make sure your user information is up-to-date by logging into Editorial Manager at Editorial Manager® and clicking the ‘Update My Information' link at the top of the page. For questions related to billing, please contact billing support.

If your institution or institutions have a press office, please notify them about your upcoming paper to help maximize its impact. If they’ll be preparing press materials, please inform our press team as soon as possible -- no later than 48 hours after receiving the formal acceptance. Your manuscript will remain under strict press embargo until 2 pm Eastern Time on the date of publication. For more information, please contact onepress@plos.org.

Kind regards,

Erfan Ghadirzadeh, MD

Academic Editor

PLOS One

Additional Editor Comments (optional):

Reviewers' comments:

Reviewer's Responses to Questions

**Comments to the Author**

1. If the authors have adequately addressed your comments raised in a previous round of review and you feel that this manuscript is now acceptable for publication, you may indicate that here to bypass the “Comments to the Author” section, enter your conflict of interest statement in the “Confidential to Editor” section, and submit your "Accept" recommendation.

Reviewer #2: All comments have been addressed

**********

2. Is the manuscript technically sound, and do the data support the conclusions?

The manuscript must describe a technically sound piece of scientific research with data that supports the conclusions. Experiments must have been conducted rigorously, with appropriate controls, replication, and sample sizes. The conclusions must be drawn appropriately based on the data presented.

Reviewer #2: Yes

**********

3. Has the statistical analysis been performed appropriately and rigorously?

Reviewer #2: N/A

**********

4. Have the authors made all data underlying the findings in their manuscript fully available?

The PLOS Data policy requires authors to make all data underlying the findings described in their manuscript fully available without restriction, with rare exception (please refer to the Data Availability Statement in the manuscript PDF file). The data should be provided as part of the manuscript or its supporting information, or deposited to a public repository. For example, in addition to summary statistics, the data points behind means, medians and variance measures should be available. If there are restrictions on publicly sharing data—e.g. participant privacy or use of data from a third party—those must be specified.

Reviewer #2: Yes

**********

5. Is the manuscript presented in an intelligible fashion and written in standard English?

PLOS ONE does not copyedit accepted manuscripts, so the language in submitted articles must be clear, correct, and unambiguous. Any typographical or grammatical errors should be corrected at revision, so please note any specific errors here.

Reviewer #2: Yes

**********

6. Review Comments to the Author

Please use the space provided to explain your answers to the questions above. You may also include additional comments for the author, including concerns about dual publication, research ethics, or publication ethics. (Please upload your review as an attachment if it exceeds 20,000 characters)

Reviewer #2: Dear Authors,

Thank you for submitting the revised version of your manuscript.

I have carefully reviewed your revised version of the manuscript and commend you on the thorough manner in which you have addressed the comments provided during the initial review. Your responses are clear, detailed, and demonstrate a strong engagement with the feedback. The revisions have notably enhanced the clarity, coherence, and overall presentation of the manuscript.

In particular, the improvements in the articulation of your findings, the strengthening of methodological transparency, and the alignment between your data and conclusions are evident. These revisions contribute positively to the manuscript’s scientific soundness and readability.

Overall, I am satisfied that the concerns raised have been adequately resolved, and the manuscript is now much improved.

Thank you for your careful and thoughtful revisions.

Kind regards.

**********

7. PLOS authors have the option to publish the peer review history of their article (what does this mean?). If published, this will include your full peer review and any attached files.

If you choose “no”, your identity will remain anonymous but your review may still be made public.

**Do you want your identity to be public for this peer review?**   For information about this choice, including consent withdrawal, please see our Privacy Policy.

Reviewer #2: **Yes:**  Adeniyi Adeleye

**********

# Editor report: Acceptance letter

**DOI:** 10.1371/journal.pone.0349948.r004

**Authors:** Erfan Ghadirzadeh

PONE-D-25-54340R1

PLOS One

Dear Dr. Rushton,

I'm pleased to inform you that your manuscript has been deemed suitable for publication in PLOS One. Congratulations! Your manuscript is now being handed over to our production team.

At this stage, our production department will prepare your paper for publication. This includes ensuring the following:

* All references, tables, and figures are properly cited

* All relevant supporting information is included in the manuscript submission,

* There are no issues that prevent the paper from being properly typeset

You will receive further instructions from the production team, including instructions on how to review your proof when it is ready. Please keep in mind that we are working through a large volume of accepted articles, so please give us a few days to review your paper and let you know the next and final steps.

Lastly, if your institution or institutions have a press office, please let them know about your upcoming paper now to help maximize its impact. If they'll be preparing press materials, please inform our press team within the next 48 hours. Your manuscript will remain under strict press embargo until 2 pm Eastern Time on the date of publication. For more information, please contact onepress@plos.org.

You will receive an invoice from PLOS for your publication fee after your manuscript has reached the completed accept phase. If you receive an email requesting payment before acceptance or for any other service, this may be a phishing scheme. Learn how to identify phishing emails and protect your accounts at https://explore.plos.org/phishing.

If we can help with anything else, please email us at customercare@plos.org.

Thank you for submitting your work to PLOS ONE and supporting open access.

Kind regards,

PLOS ONE Editorial Office Staff

on behalf of

Dr. Erfan Ghadirzadeh

Academic Editor

PLOS One
